# Vision-Based Leader Vehicle Trajectory Tracking for Multiple Agricultural Vehicles

**DOI:** 10.3390/s16040578

**Published:** 2016-04-22

**Authors:** Linhuan Zhang, Tofael Ahamed, Yan Zhang, Pengbo Gao, Tomohiro Takigawa

**Affiliations:** Graduate School of Life and Environmental Sciences, University of Tsukuba, 1-1-1 Tennodai, Tsukuba 305-8572, Japan; zhanglinhuanch@yahoo.co.jp (L.Z.); yanzhang810@hotmail.com (Y.Z.); pengbogao@outlook.com (P.G.)

**Keywords:** multiple vehicles, monocular vision, quadratic curve fitting, trajectory tracking

## Abstract

The aim of this study was to design a navigation system composed of a human-controlled leader vehicle and a follower vehicle. The follower vehicle automatically tracks the leader vehicle. With such a system, a human driver can control two vehicles efficiently in agricultural operations. The tracking system was developed for the leader and the follower vehicle, and control of the follower was performed using a camera vision system. A stable and accurate monocular vision-based sensing system was designed, consisting of a camera and rectangular markers. Noise in the data acquisition was reduced by using the least-squares method. A feedback control algorithm was used to allow the follower vehicle to track the trajectory of the leader vehicle. A proportional–integral–derivative (PID) controller was introduced to maintain the required distance between the leader and the follower vehicle. Field experiments were conducted to evaluate the sensing and tracking performances of the leader-follower system while the leader vehicle was driven at an average speed of 0.3 m/s. In the case of linear trajectory tracking, the RMS errors were 6.5 cm, 8.9 cm and 16.4 cm for straight, turning and zigzag paths, respectively. Again, for parallel trajectory tracking, the root mean square (RMS) errors were found to be 7.1 cm, 14.6 cm and 14.0 cm for straight, turning and zigzag paths, respectively. The navigation performances indicated that the autonomous follower vehicle was able to follow the leader vehicle, and the tracking accuracy was found to be satisfactory. Therefore, the developed leader-follower system can be implemented for the harvesting of grains, using a combine as the leader and an unloader as the autonomous follower vehicle.

## 1. Introduction

Multiple autonomous vehicles can improve the efficiency of agricultural operations by performing labor-intensive tasks such as transporting, plowing, sowing, fertilizing, spraying, and harvesting [1,2]. The simultaneous control of multiple robotic vehicles has received attention from several researchers. For example, multiple moss-harvesting robotic tractors were commanded and monitored by a human driver, who also functioned as the leader [3]. To enable navigation under complex road conditions, an autonomous follower tractor could change formation with the human-driven leader tractor to avoid obstacles based on commands from the leader [4]. The FOLLOW and GOTO algorithms were developed to control multiple vehicles in a flexible way, both in formation and independently [5].When considering the farming task style, a common operational method of multiple autonomous vehicles should be effective when an autonomous or a human-driven leader vehicle can lead one or more follower vehicles. Furthermore, following a trajectory identical or parallel to that of the leader is important in outdoor farm conditions. For example, while driving along a narrow road with obstacles, followers can adopt an in-line formation with the leader for safety, whereas while working on farmland, followers in a parallel formation with the leader could follow trajectories parallel to that of the leader, allowing the farming task to be performed without overlap or missed areas. For such a target, integrity model involving leader motion information, such as steering and the speed of the leader, could allow for precise tracking [6,7,8]. However, the transmission of the leader motion information to the follower through a wireless device creates the risk of wireless distribution or failure. Aiming to solve this problem, the leader’s position and velocity were estimated based on local sensors [9], and a neural network (NN)-based extended Kalman filter (EKF) was designed to estimate leader speed and accommodate modeling errors [10]. By cooperating with GPS location, a time-delayed leader tracking model was established and showed excellent tracking performance [11].

In any event, the follower vehicle needs to continually update its relative position with respect to the leader to fulfill the tracking task. Regarding safety, absolute sensors such as those employing GPS are not suitable for the tracking task because they may lose the satellite signal and are subject to multipath interference. Local sensors, such as cameras and laser range finders (LRF), are considered to be better approaches and have been successfully applied for tracking under both indoor and outdoor conditions [12,13,14,15]. Compared with LRFs, camera vision can provide more information than data obtained via LRF scanning with less cost and has thus been wildly utilized for navigation, mapping and tracking [16,17,18]. For tracking control of multiple robotic vehicles, a camera vision-based leader-follower relative position estimating method has been designed, recognizing a leader vehicle using features of the leader vehicle [19,20]. However, this method was sensitive to lighting conditions and was distance-limited as well as time-intensive. A common and effective method for solving those problems was to use an artificial marker to identify the leader and estimate the leader-follower relative position using pre-known geometry or color information of the markers [21]. The advantages of the marker-based method are that it could support stable recognition, accurate position estimation, and fast calculation. Moreover, it ensures the tracking accuracy and tracking safety for a leader-follower trajectory tracking system. In this research, to avoid using GPS and wireless devices, the designed control law of the follower vehicle for leader trajectory tracking only relied on the relative leader-follower position, which was obtained from the camera vision. This study aimed to develop a vision-based feedback controller designed to track a leader-vehicle trajectory while maintaining an in-line or parallel formation. Thus, the objectives of this research were as follows:

(1) To establish an autonomous vehicle as a follower vehicle able to conduct tracking tasks.
(2) To construct a robust and accurate monocular vision system able to estimate the relative position between a leader and a follower.
(3) To develop a control algorithm able to realize accurate leader vehicle trajectory-tracking for multiple agricultural machinery combinations, with a human-driven leader and an autonomous follower.

## 2. Materials and Methods

**Figure 1:**
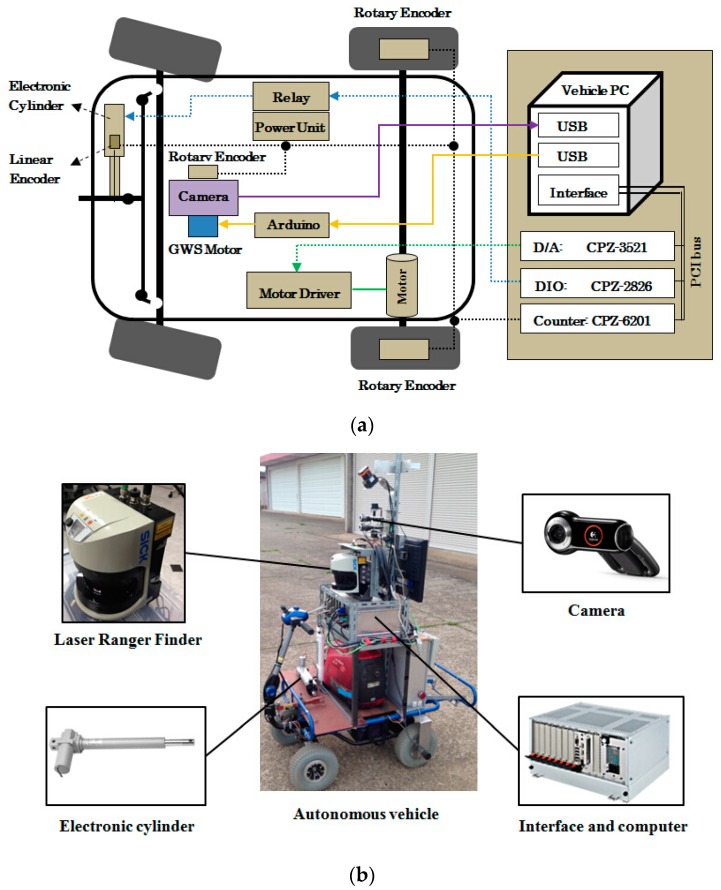
The autonomous follower in the leader-follower system. (**a**) Sensors arrangements in the autonomous unit; (**b**) Hardware components of the autonomous follower tracking system.

An electronic vehicle (CHIKUSUI EJ-20, CANYCOM, Tokyo, Japan) was modified into an autonomous vehicle, *i.e.*, the follower vehicle. Both the leader and the follower vehicle had a 60 cm wheel base length and 49 cm drawbar length. Major subsystems of the autonomous vehicle included steering control, speed control, power and sensor units. The sensory data and control status were transmitted to an upper level controller through parallel communication. The basic instrumentation system for the autonomous vehicle is depicted in Figure 1a. A Pro 9000 Web camera (Logitech, Lausanne, Switzerland) with 2 million pixels and a 70° view angle, was mounted on the autonomous follower vehicle to provide vision information. A LMS 511 LRF (SICK, Waldkirch, Germany) was utilized as an assist device to provide reference data and recorded trajectories during tracking. Steering control of the autonomous vehicle was conducted using an electronic cylinder (LPF040L2.0VK2J, TSUBAKIMOTO CHAIN, Osaka, Japan). The length of the piston rod was 200 mm and the maximum speed was 40 mm/s. It could provide stable thrust power up to 400 N. Figure 1b shows components of the robot vehicle.

### 2.1. Leader-Follower Relative Position and Camera-Marker Sensing System

**Figure 2:**
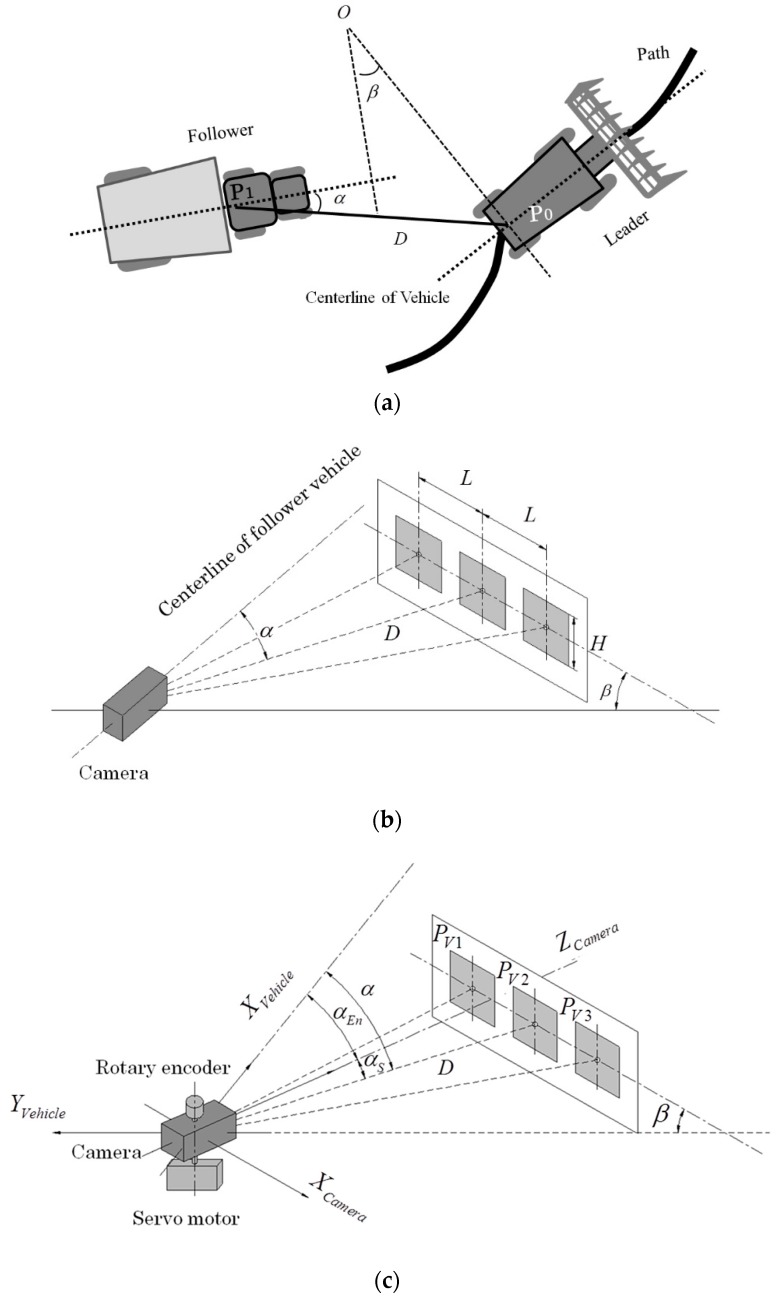
Geometrical disposition between the leader and the follower. (**a**) Leader-follower relative position; (**b**) Relative position between camera and marker plane; (**c**). Servo motor implemented with the camera-marker system.

Figure 2a describes the relative position between the leader and follower. By identifying the relative heading angle β, relative distance *D*, and orientation angle α of the leader relative to the follower, the follower vehicle could identify the leader position. The leader-follower relative position was obtained from the camera-marker system (Figure 2b), in which the camera was mounted on the rear wheel center point $P_{1}$ of the follower vehicle, the marker was installed perpendicular to the centerline of the leader vehicle and the position of the middle square of the marker was at the rear wheel center point $P_{0}$. The side length of each square *H* and the interval between square centers *L* were 0.2 m and 0.4 m, respectively. In the leader-follower system, the following steps were followed to develop the relative positioning system: camera servo systems, marker detection, marker positioning, and estimation of offset of the roll angle between camera and marker.

#### 2.1.1. Camera Servo System

Losing the target was a severe problem during the tracking of the leader vehicle; it potentially occurred owing to the limitations of the camera view field, especially on a large-curvature path. To overcome this problem, a camera servo system was designed to keep the marker in the center of the camera view field. The camera servo system comprised a GWS servo motor and a rotary encoder with a camera (Figure 2c). By responding to the angle $\alpha_{s}$ from the middle square center to the camera optical axis, the servo motor could rotate the camera directly to the marker center. The rotation angle $\alpha_{En}$ between the optical axis and the centerline of the follower vehicle could be monitored by a rotary encoder installed above the camera. The existing follow relationship can be expressed as:

(1) $$\alpha =\alpha_{s}+\alpha_{En}$$

#### 2.1.2. Marker Detection

**Figure 3:**
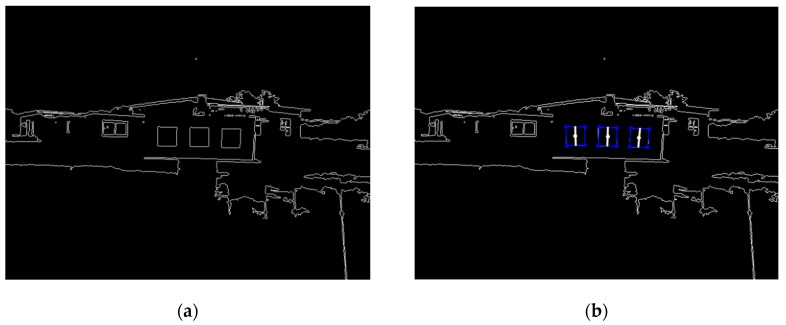
Image processing for marker detection. (**a**) Contour image; (**b**) Detected marker.

The marker was detected based on its pre-known geometry information, including its square shape features and relative spatial relationship between squares in the marker plane. The image processing flow comprises the following four steps: transforming an original RGB image into a grayscale image and then enhancing the contrast ratio, extracting contours, finding rectangles from the contour images, and determining the marker (Figure 3).

Pre-known geometry information could reduce the computational cost and benefit real-time detection. Additionally, the high contrast ratio between the black squares and the white background enabled the generation of acutance contours and created stability for the detection of the marker. However, contour extraction was still influenced by illumination conditions. Low illumination conditions or strong sunlight under an outdoor environment would reduce the contrast ratio of the image and corrode the contour of squares, causing failure of marker detection. To expand the scope to adapt to various illumination conditions, a commonly used normal distribution of the image histogram method was utilized to enhance image contrast. Affected by posture changes of the vehicles, squares projected on the image plane would show the shapes of rectangles. Thus, rectangles were recognized and selected in the contour image. Relying on the relative spatial relationship between the three squares, false targets with rectangular shapes, such as rooms and windows, could be filtered, and only squares formed by the marker could be extracted.

#### 2.1.3. Marker Positioning

**Figure 4:**
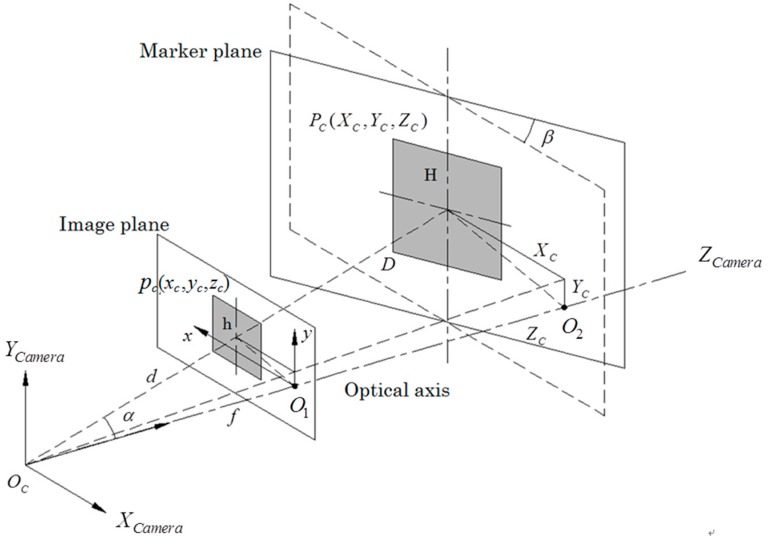
Camera perspective model.

Given that the vision data were obtained from a single camera and the relative position between the marker and the camera was estimated based on the known side length of the marker squares, the position of each square in the marker plane could be described by its center point. The pitch angle of the vehicle body was neglected, meaning that the sides of squares in the vertical direction would not be affected by the posture changes of the leader and follower vehicles when projected onto the image plane. For this reason, the centerline of the squares in the vertical direction could be used to estimate the relative position between the camera and marker plane. Utilizing the geometric relationship between similar triangles under a perspective model (Figure 4), the position of the square center $P_{C}$ in camera-based coordinates could be estimated as follows:

(2) $$X_{C}=\frac{x-c_{x}}{f_{x}}Z_{C}$$

(3) $$Z_{C}=\frac{H}{h}f$$

(4) $$\alpha =\text{arctan}\left(\frac{X_{C}}{Z_{C}}\right)$$

where the coordinates of the square center under the image coordinate and camera-based coordinate systems could be written as $p_{c}\left(x_{c},y_{c},f\right)$ and $P_{C}\left(X_{C},Y_{C},Z_{C}\right)$, respectively. f and $f_{x}$ represent the focal length, and $c_{x}$ is the shift of the optical axis obtained from camera calibration; *h* is the height of squares in the image plane.

#### 2.1.4. Offset of Roll Angle between Camera and Marker

**Figure 5:**
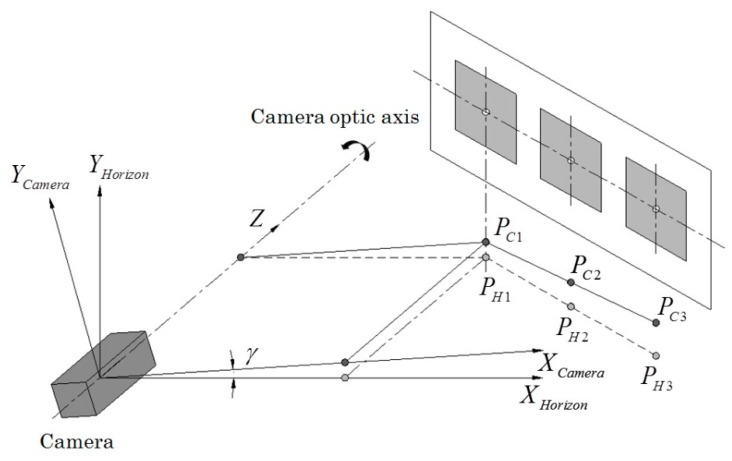
Model for offsetting vehicle roll effect.

On uneven farm ground, rolling of the camera or the marker plane would occur and affect the leader-follower relative position observation accuracy. The calculation of the leader-follower relative position should offset the rolling effect of the camera or the marker plane. For example, suppose the leader vehicle is driven on a horizontal surface, while the follower vehicle forms a roll angle γ around its optical axis from the horizontal surface (Figure 5). $P_{CN}\left(X_{CN},Y_{CN},Z_{CN}\right)$ are the coordinates of square centers based on the camera coordinate system and $P_{HN}\left(X_{HN},Y_{HN},Z_{HN}\right)$ are the coordinates of square centers with respect to the horizontal surface (Figure 5). Clearly, the position of $P_{CN}$ represents the relative position between the camera and the marker plane, and the position $P_{HN}$ represents the relative position between the follower and the leader vehicles. Thus, the relationship between $P_{CN}$ and $P_{HN}$ could be written as:

(5) $$X_{CN}=X_{HN}\cos\gamma -Y_{HN}\sin\gamma$$

(6) $$Z_{CN}=Z_{HN}$$

Because the relative position between the leader and the follower vehicle only corresponds to the *X-Z* coordinates, the square centers can be assumed to lie on the horizontal surface. Then, Equation (5) can be rewritten as:

(7) $$X_{HN}=\frac{X_{CN}}{\cos\gamma }$$

(8) $$\gamma =tan^{-1}\left(\frac{3\sum_{n=1}^{3}x_{cn}y_{cn}-\left(\sum_{n=1}^{3}x_{cn}\right)\left(\sum_{n=1}^{3}y_{cn}\right)}{3\sum_{n=1}^{3}x_{cn}^{2}-{\left(\sum_{n=1}^{3}x_{cn}\right)}^{2}}\right)$$

where $\left(x_{cn},y_{cn}\right)$ represent coordinates of the square centers $p_{c}\left(x_{c},y_{c},f\right)$ in the plane.

#### 2.1.5. Transformation of Coordinates and Relative Positioning of the Marker

The transformation of coordinates between the camera and the follower vehicle could be expressed as follows (Figure 2c):

(9) $$X_{VN}=\frac{X_{CN}}{\cos\gamma }\sin\alpha_{En}+Z_{CN}\cos\alpha_{En}$$

(10) $$Y_{VN}=\frac{X_{CN}}{\cos\gamma }\cos\alpha_{En}+Z_{CN}\sin\alpha_{En}$$

where $P_{VN}\left(X_{VN},Y_{VN}\right)$ are the coordinates of the square centers in the follower-based local coordinates. The relative distance *D* and relative angle β between the leader and the follower vehicle could be calculated as:

(11) $$D=\sqrt{X_{V2}^{2}+Y_{V2}^{2}}$$

(12) $$\beta =tan^{-1}\left(\frac{3\sum_{N=1}^{3}X_{VN}Y_{VN}-\left(\sum_{N=1}^{3}X_{VN}\right)\left(\sum_{N=1}^{3}Y_{VN}\right)}{3\sum_{N=1}^{3}X_{VN}^{2}-{\left(\sum_{N=1}^{3}X_{VN}\right)}^{2}}\right)$$

Then, the relative position between the leader and the follower vehicle could be written as

(13) $$x_{l\_F}=X_{V2}$$

(14) $$y_{l\_F}=Y_{V2}$$

(15) $$\theta_{l\_F}=\beta$$

where $x_{l\_F}$, $y_{l\_F}$ represents the local position of the leader based on the follower and $\theta_{l\_F}$ is the local heading angle of the leader based on the follower. 

### 2.2. Camera Vision Data Estimation and Smoothing

Limited by the monocular vision method, the observed leader-follower relative position was noisy under the worst farm conditions. In some cases, large observed errors would occur or there was even a failure to detect the marker plane. The estimation and smoothing of the observation data were necessary to ensure the accurate tracking of the leader vehicle and also to improve the motion stability of the follower vehicle. Because the motion of the two vehicles was continuous, the variation of relative distance and angle between the leader and the follower vehicle was also continuous. The commonly used method of least-squares was introduced to estimate and smooth the relative distance *D* and the relative heading angle β between the leader and the follower vehicle by fitting a quadratic curve separately. During the process of data estimation and smoothing, estimated data could be obtained by fitting the stored latest *n* points of observation data to a quadratic curve using the least-squares method. In this study, the quadratic curve could be written as:

(16) $$q\left(n\right)=an^{2}+bn+c$$

where *n* denotes observation times used to store and fit the data, and q(n) is the vector of the stored observation data sequence, including the relative distance and the relative heading angle. q(n) is defined as:

(17) $$q\left(n\right)=\left(\begin{array}{c} D\left(n\right)\\ \beta \left(n\right) \end{array}\right)$$

To ensure the fitting effect, avoid collapse of the least-squares method and maintain the original transfer tendency of the leader-follower relative position, the data stored for fitting required appropriate handling. The estimation and smoothing process was realized through two steps: first, once a new camera observation was available, the sequence of the stored observation data would be updated and the latest stored data after updating was temporarily determined as follows:

(18) q(i)=q(i+1)   i∈(0,1……n−1)

(19) $$q\left(n\right)=\{\begin{array}{c} q_{C\_obs}\\ q\left(n-1\right) \end{array}\;\begin{array}{c} q_{E\_1}<q_{Th}\\ q_{E\_1}>q_{Th} \end{array}$$

(20) $$q_{E\_1}=\left|q\left(n-1\right)-q_{C\_obs}\right|$$

Second, after fitting to the quadratic curve using the least-squares method, the latest stored data and the current leader-follower relative position could be determined as follows:

(21) $$q\left(n\right)=\{\begin{array}{c} q_{C\_obs}\\ q_{Fit} \end{array}\;\begin{array}{c} q_{E\_2}<q_{Th}\\ q_{E\_2}>q_{Th} \end{array}$$

(22) $$q_{E\_2}=\left|q_{C\_obs}-q_{Fit}\right|$$

(23) $$q_{Est}=q\left(n\right)$$

where $q_{C\_obs}$ is the vector of the current camera observed data, $q_{Fit}$ is the vector of the fitted current relative distance and relative angle using the stored *n* times of observation data, $q_{Est}$ is the vector defining the current relative distance and relative angle, q(i) is the vector of the stored $i_{th}$ observation, $q_{E\_1}$ is the vector of the distance between the current observation and last observation, $q_{E\_2}$ is the vector of the distance between the current observation and fitted observation, and $q_{Th}$ is the vector of the threshold values, set as (1 m, 40°). 

### 2.3. Design of Control Law for the Leader Trajectory Tracking of the Follower Vehicle

In this study, only the leader-follower relative position information was used by the follower vehicle to track the leader trajectory. The absence of information exchange and absolute reference positions made the leader trajectory thoroughly uncertain for the follower vehicle, and the tracking position for the follower vehicle was ambiguous. A feedback control method based on the leader-follower relative position was proposed to track the trajectory of the leader. 

**Figure 6:**
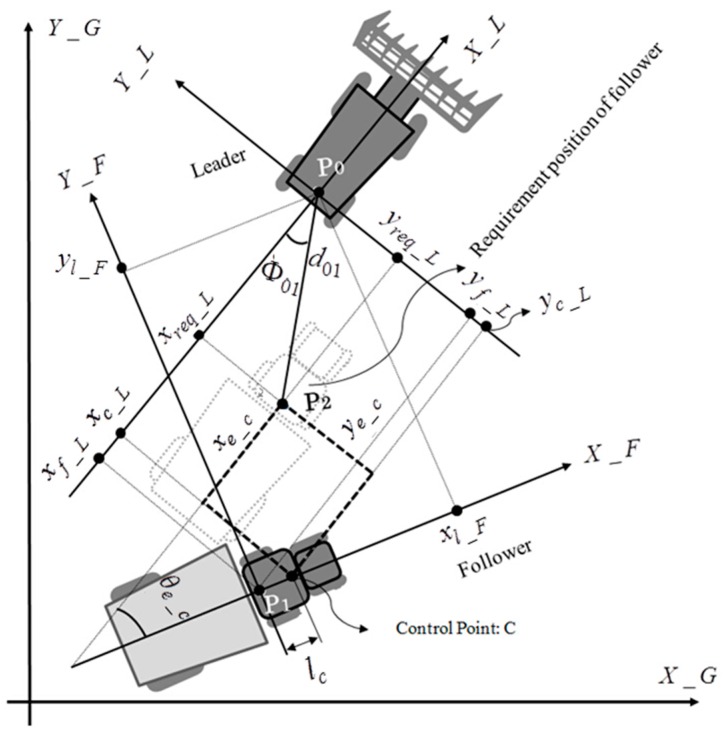
Relationship and coordinate transformation between the leader and the follower vehicles.

As described in Figure 6, the required position of the follower vehicle is set at $P_{2}$, with a distance $d_{01}$ from the leader vehicle rear axis and an angle $\Phi_{01}$ with the leader vehicle rear axis. Assuming the leader vehicle is driven with a straight trajectory, the position of $P_{2}$ in the leader-based local coordinates could be written as:

(24) $$\left[\begin{array}{c} x_{req\_L}\\ y_{req\_L}\\ \theta_{req\_L} \end{array}\right]=\left[\begin{array}{c} d_{01}cos\;\Phi_{01}\\ d_{01}sin\;\Phi_{01}\\ 0 \end{array}\right]$$

To improve the control freedom of the follower vehicle to realize the tracking of the uncertain leader vehicle trajectory, a control point *C*, located on the centerline of the follower vehicle, was introduced. Moreover, the distance from the rear wheel axial center to the control point *C* was defined as:

(25) $$l_{c}=k_{0}l$$

where *l* is the length from the front wheel axial center to the rear wheel axial center; $l_{c}$ is the length from the rear wheel axial center to the control point *C*; the parameter $k_{0}$ is used to determine the location of control point *C*. The position of the control point *C* in leader-based local coordinates could be written as:

(26) $$\left[\begin{array}{c} x_{c\_L}\\ y_{c\_L}\\ \theta_{c\_L} \end{array}\right]=-\left[\begin{array}{ccc} cos\;\beta & sin\;\beta & 0\\ -sin\;\beta & cos\;\beta & 0\\ 0 & 0 & 1 \end{array}\right]\left[\begin{array}{c} x_{l\_F}\\ y_{l\_F}\\ \theta_{l\_F} \end{array}\right]+\left[\begin{array}{c} l_{c}\;cos\;\beta \\ -l_{c}\;sin\;\beta \\ 0 \end{array}\right]$$

Combined with Equations (13)–(15) and (24)–(26), the control point *C*-based position tracking error between the follower vehicle and its requirement position could be calculated as:

(27) $$\left[\begin{array}{c} x_{e\_c}\\ y_{e\_c}\\ \theta_{e\_c} \end{array}\right]=\left[\begin{array}{c} x_{c\_L}\\ y_{c\_L}\\ \theta_{c\_L} \end{array}\right]-\left[\begin{array}{c} x_{req\_L}\\ y_{req\_L}\\ \theta_{req\_L} \end{array}\right]=-\left[\begin{array}{ccc} cos\;\beta & sin\;\beta & 0\\ -sin\;\beta & cos\;\beta & 0\\ 0 & 0 & 1 \end{array}\right]\left[\begin{array}{c} X_{V2}\\ Y_{V2}\\ \beta \end{array}\right]+\left[\begin{array}{c} l_{c}\;cos\;\beta \\ -l_{c}\;sin\;\beta \\ 0 \end{array}\right]-\left[\begin{array}{c} d_{01}cos\;\Phi_{01}\\ d_{01}sin\;\Phi_{01}\\ 0 \end{array}\right]$$

A simple steering strategy for responding to longitudinal and heading tracking error is given as:

(28) $$\delta =k_{1}y_{e\_c}+k_{2}\theta_{e\_c}+k_{3}\;sin\;\theta_{e\_c}$$

A PID controller was designed to maintain the required distance between the leader and the follower vehicle; control of the follower velocity could be given as:

(29) $$v_{t}=v_{t-1}+k_{D}\left(e_{t}-e_{t-1}\right)+k_{I}e_{t}+k_{P}\left(e_{t}-2e_{t-1}+e_{t-2}\right)$$

(30) $$e=\left(D-d_{01}\right)$$

where $k_{1}$, $k_{2}$, $k_{3}$ are control parameters corresponding to the required distance $d_{01}$ and angle $\Phi_{01}$; $k_{D}$, $k_{I}$, $k_{P}$ are parameters of the PID controller adjusted during field experiments. Notice that once the required values of distance $d_{01}$ and angle $\Phi_{01}$ were altered, the control parameters also needed to be adjusted.

## 3. Field Experiments

**Figure 7:**
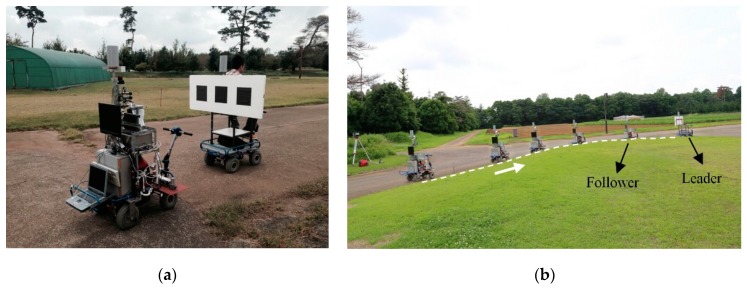
Field experiments of the leader-follower system. (**a**) Evaluation of the camera–marker system; (**b**) Tracking of a trajectory of the leader vehicle.

Experiments for verifying the stability and accuracy of the camera-marker sensing system and leader trajectory tracking accuracy were conducted at the Agricultural and Forestry Research Center, University of Tsukuba (Ibaraki, Japan). The camera-marker sensing system evaluation experiments included both a static and a dynamic evaluation experiment. The static evaluation experiment was intended to verify the stability and accuracy of the designed observation method and optimize the camera coefficients. The dynamic evaluation experiment was designed to determine the threshold values for data estimation and smoothing, analyze the observation stability and accuracy, and verify the effectiveness of the least-squares method-based data estimation and smoothing solution. A SICK LMS 511 LRF was used to provide reference data, and the relative position from the LRF to the marker plane was used as reference data to evaluate the camera observation accuracy (Figure 7a). 

In the tracking accuracy evaluation experiments, linear and parallel tracking experiments were conducted on straight, turning, and zigzag paths. Cylindrical markers were mounted above the rear wheel centers of the leader and follower vehicles (Figure 7b) to facilitate the LMS 511 LRF in recording their trajectories at a frequency of 25 Hz. The leader vehicle was driven at a velocity of 0.3 m/s. The required distance $d_{01}$ between the leader and the follower vehicle was 4 m in linear tracking. In parallel tracking, the required lateral and longitudinal offsets of the follower vehicle were set at 4 m and 2 m from the leader vehicle so that the trajectory of the follower vehicle could parallel that of the leader vehicle at a 2 m interval. 

## 4. Results 

### 4.1. Evaluation of Camera-Marker Observation System

**Figure 8:**
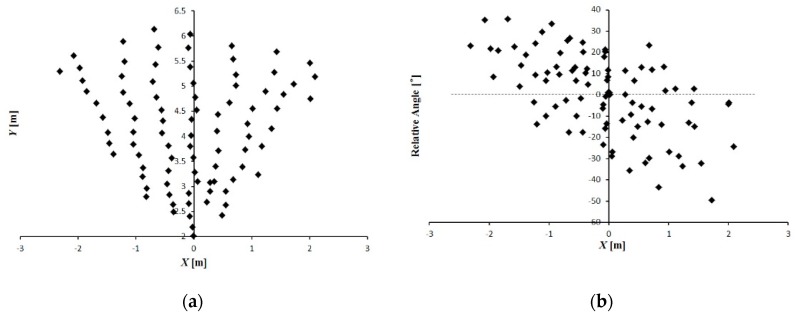
Position of the marker. (**a**) Location of the marker; (**b**) Relative angle between the marker and the x-axis.

**Figure 9:**
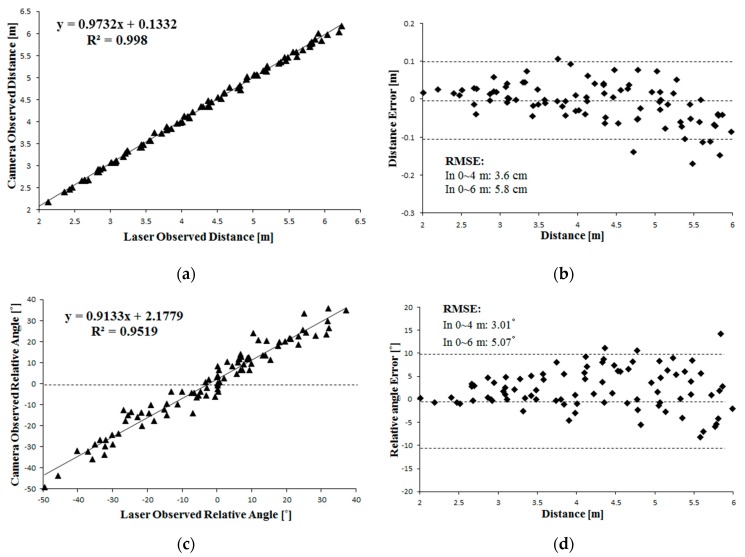

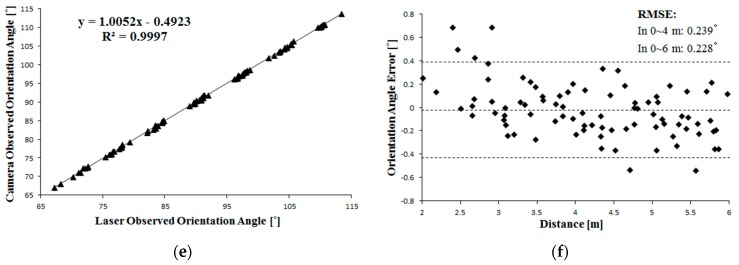
Linear Regression and Accuracy analysis of the camera observation referenced with the laser observation. (**a**) Distance; (**b**) Relative angle; (**c**) Orientation angle; (**d**) Distance error; (**e**) Relative angle error; (**f**) Orientation angle error.

In the static evaluation experiment, the maximum distance from the camera to the marker was approximately 6 m and the relative angle that formed between the marker and the camera axis ranged from −40° to 40° (Figure 8). Using the LRF data as reference, the accuracy of the leader-follower relative position obtained from the camera-marker system could be evaluated (Figure 9). Linear regression analysis showed that the orientation angle and distance between the leader and follower vehicles obtained from the camera-marker system were stable and had high accuracy (Figure 9a,e). Meanwhile, the leader-follower relative angle obtained from the camera-marker system was unstable (Figure 9c). Compared with the accuracy of the orientation angle (Figure 9f), the accuracy of the distance and relative angle obtained from the camera-marker system degraded as the relative distance from the camera to the marker increased (Figure 9b,d). This phenomenon was mainly caused by the limitation of the camera; the pitch angle of the vehicle also potentially caused an observation error of the uneven ground. 

The RMS errors of the leader-follower relative distance, relative angle, and orientation angle observation were calculated. When the distance between the camera and the marker was 6 m, the RMS errors of the leader-follower relative distance, relative angle and orientation angle observation were 5.8 cm, 5.07° and 0.228°, respectively. At 4 m, the RMS errors of the leader-follower relative distance, relative angle and orientation angle observation were 3.63 cm, 3.01° and 0.239°, respectively. Considering that the orientation angle obtained from the camera-marker system was stable and had high accuracy, data estimation and smoothing was only conducted for the distance and relative angle observed. In the dynamic evaluation experiment, the leader vehicle was driven along a zigzag path and the follower vehicle was controlled in remote mode to follow the leader. The camera observation data before estimation and smoothing, the estimated and smoothed data obtained through least-squares-based curve fitting, and the LRF observation data were recorded during driving.

**Figure 10:**
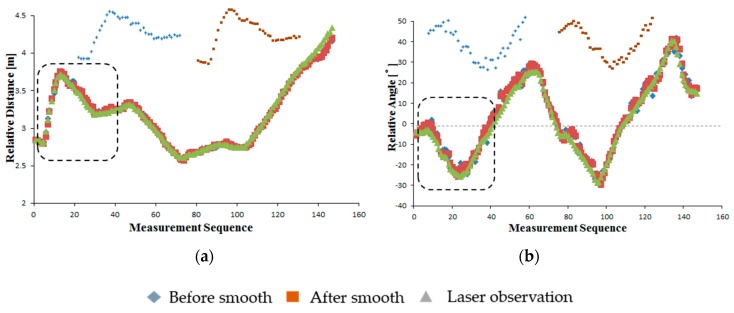
Relative position between the camera and marker before smooth, smoothed, and LRF data. (**a**) Relative distance; (**b**) Relative angle.

**Figure 11:**
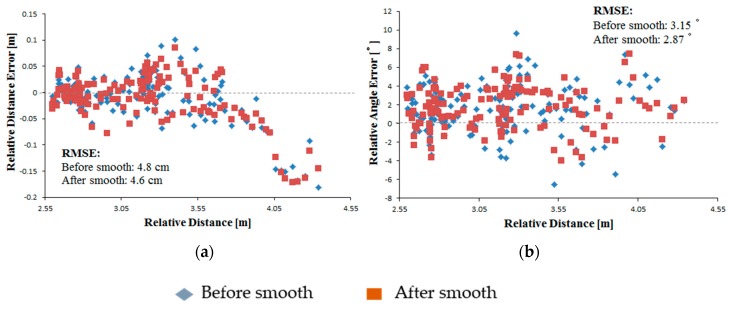
Relative position error for camera observation. (**a**) Relative distance error; (**b**) Relative angle error.

**Figure 12:**
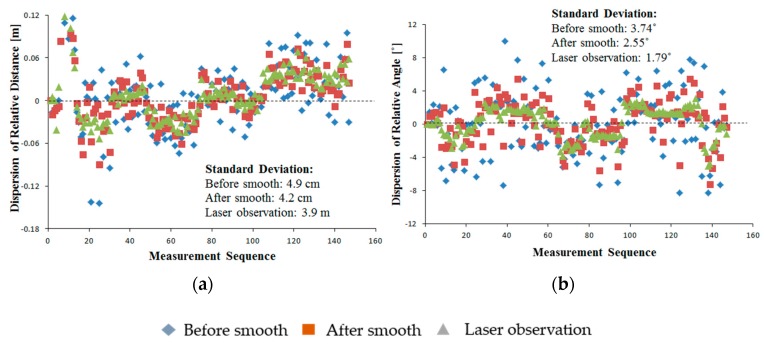
Dispersion of camera observation data. (**a**) Dispersion of relative distance; (**b**) Dispersion of relative angle.

The results showed that both the camera data before estimation and smoothing and the estimated and smoothed camera data closely matched the LRF data (Figure 10). The RMS errors of the camera observation before estimation and smoothing were 4.7 cm and 3.15° for the relative distance and relative angle, respectively. These coincided with the results under static conditions, meaning that the motion of the marker and the camera had little effect on the observation accuracy. During the experiment, the camera-observed data were smoothed by fitting a curve using the least-squares method. After data estimation and smoothing, the camera observation data were observably smoothed, as shown in the dotted rectangle (Figure 10). Furthermore, the accuracy of the leader-follower relative position observation was improved after data estimation and smoothing, and the RMS errors of the relative distance and relative angle were reduced to 4.6 cm and 2.87°, respectively (Figure 11). Compared with the camera observation data before estimation and smoothing, the dispersion of the estimated and smoothed data were also reduced, with the standard deviations of the relative distance and relative angle reduced from 4.9 to 4.2 cm and 3.74 to 2.55°, respectively (Figure 12). Those performances showed the potential for stable and accurate observation when applied to real-sized tractors, being clearly insensitive to the uneven ground and having stable motion characteristics compared with the small-sized vehicles.

### 4.2. Tracking Performance

**Figure 13:**
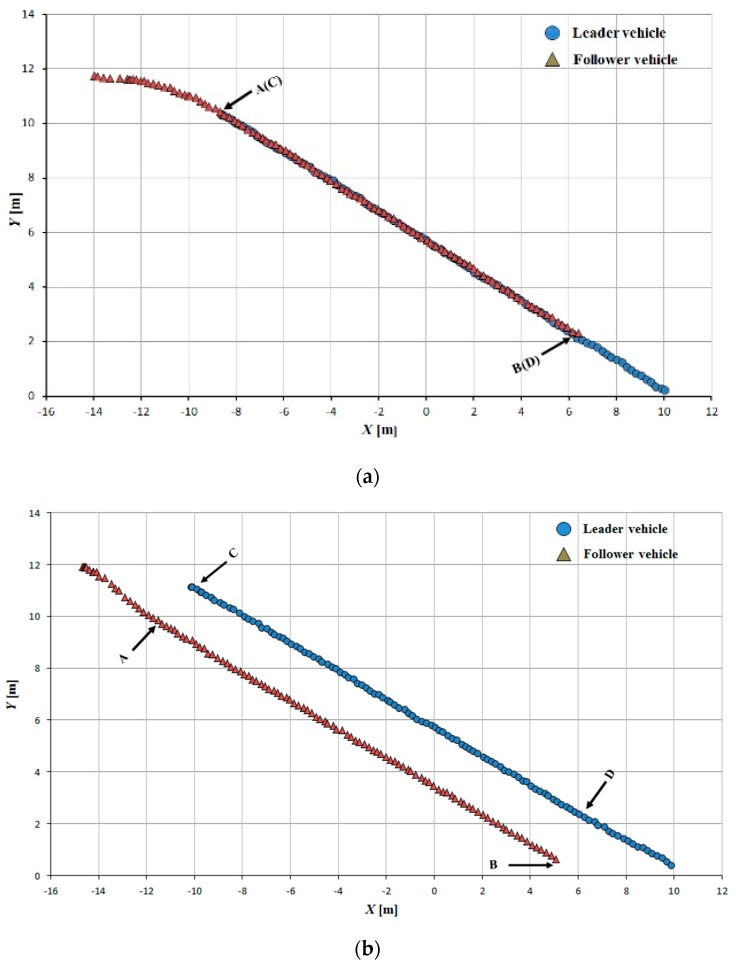
Leader trajectory tracking on a straight path. (**a**)Linear tracking; (**b**) Parallel tracking.

**Figure 14:**
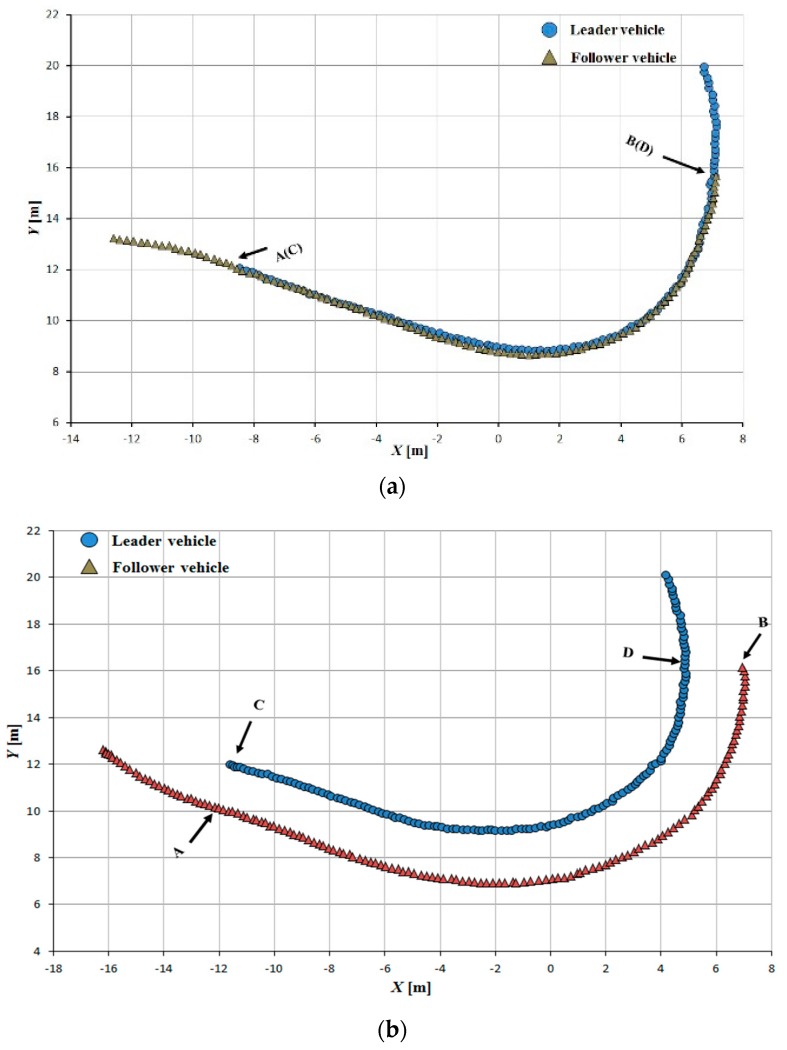
Leader trajectory tracking on a turning path. (**a**)Linear tracking; (**b**) Parallel tracking.

**Figure 15:**
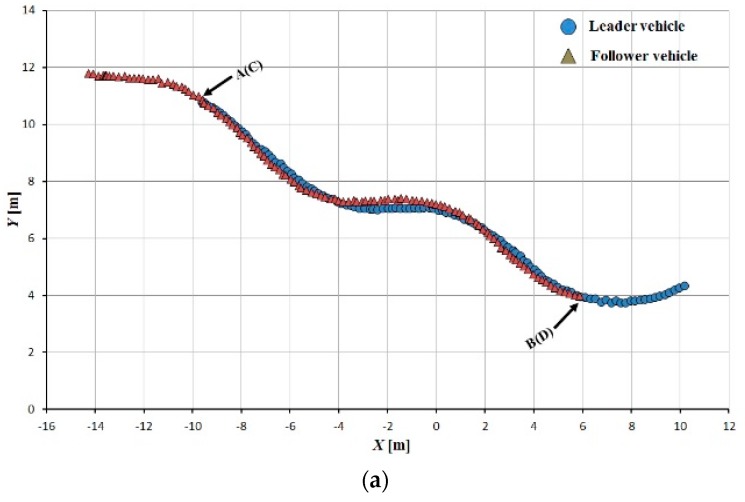

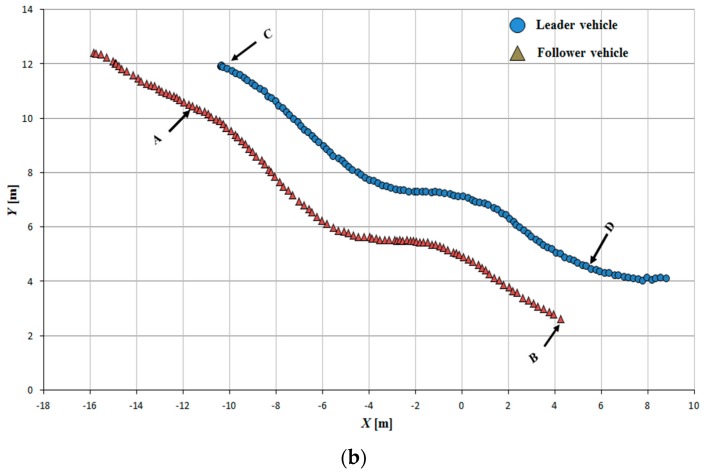
Leader trajectory tracking on a zigzag path. (**a**)Linear tracking; (**b**) Parallel tracking.

Tracking accuracy was evaluated using the interval space between the leader and follower vehicle trajectories; the trajectory segments AB and CD were used to calculate this interval space. The follower vehicle could adjust its state and arrive at its required position relative to the leader rapidly and smoothly (Figure 13, Figure 14 and Figure 15).

**Figure 16:**
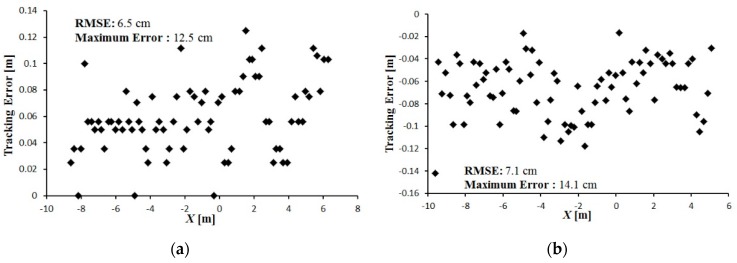
Tracking error between leader and the follower trajectories during tracking on a straight path. (**a**) Linear tracking; (**b**) Parallel tracking.

**Figure 17:**
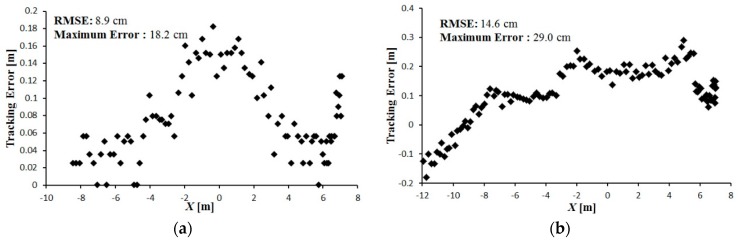
Tracking error between the leader and the follower trajectories during tracking on a turning path. (**a**) Linear tracking; (**b**) Parallel tracking.

**Figure 18:**
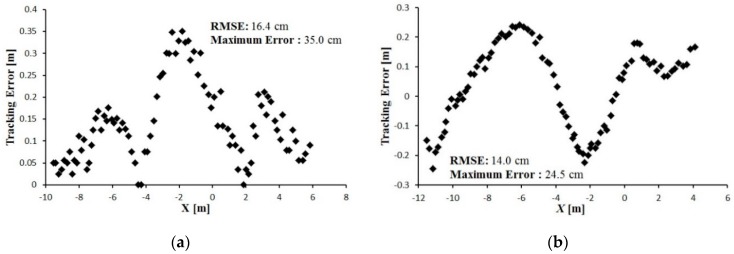
Tracking error between the leader and the follower trajectory during tracking on a zigzag path. (**a**) Linear tracking; (**b**) Parallel tracking.

The tracking error between the leader and follower vehicle trajectories is shown in Figure 16, Figure 17 and Figure 18. During tracking on a straight path, a very low tracking error between the trajectories of the leader and follower vehicles was observed; the maximum and RMS tracking errors between these trajectories were 12.5 and 6.5 cm for linear tracking and 14.1 and 7.1 cm for parallel tracking, respectively (Figure 16). During tracking on a turning path, the maximum and RMS tracking errors between the trajectories were 18.2 cm and 8.9 cm for linear and 29.0 cm and 14.6 cm for parallel tracking, respectively (Figure 17). During tracking on a zigzag path, the maximum and RMS tracking errors between the trajectories were 35.0 cm and 16.4 cm for linear and 24.5 cm and 14.0 cm for parallel tracking, respectively (Figure 18). In comparison with the straight path, the turning and zigzag path tracking showed higher error. From trajectories of the leader and follower vehicles, it can be observed that a larger variation of the direction of the leader vehicle would result in a larger tracking error (Figure 14 and Figure 15). This error remained at a low level when the leader vehicle was driven on a constant-curvature path. Considering road space and agricultural operations, the tracking accuracy was sufficient to ensure safe tracking and precision operation. 

## 5. Discussion

The driverless follower is the key of this research, which confirmed the high accuracy in following the human driven leader and the performance of the control system. Experiments were conducted using a specially built robot as follower to confirm the accuracy of tracking and develop a control system without any built-in communication between the leader and the follower. The travelling courses were chosen according to standard agricultural operations, such as straight, turning and zigzag paths. The tracking performance between the leader and the follower was satisfactory under regular field conditions. Undulating terrain and adverse climatic conditions were ignored in the field experiments. The experiments were conducted mostly under daytime conditions. The camera marker system was assisted with the LRF for cross checking the accuracy of the marker positions both in static and dynamic conditions. The LRF was utilized as an assist device to provide reference data and recorded the trajectories during tracking. The contour extraction was influenced by illumination conditions. Low illumination conditions or strong sunlight under an outdoor environment would reduce the contrast ratio of the image and affect the contour of squares, causing marker detection failure. To expand the scope to adapt to various illumination conditions, the image contrast was enhanced using a histogram method that ensured stable observation under various light conditions while conducting experiments during the daytime. The vertical vehicle’s movement or pitch angle of the vehicle body was not considered, as there was not much effect of posture changes of the leader and follower vehicles when projected onto the image plane while travelling on regular ground. This was one of the limitations of this research. However, to overcome such limitations, the centerline of the squares in the vertical direction was used to estimate the relative position between the camera and marker plane. The experiments were conducted with the prototype robot to confirm the accuracy and develop suitable control systems. The validation was done with a human driven small vehicle as leader and the autonomous prototype robot as follower. Definitely in an agricultural environment an actual size autonomous unit can be used as follower by implementing the proposed camera marker sensing and control systems. The productivity would definitely be higher, by reducing labor through enabling the human driven leader and autonomous follower system. 

## 6. Conclusions

In this study, a human-driven leader and automatic follower trajectory-tracking system was developed. A low cost camera servo system, comprising a web camera, encoder and a servomotor, was implemented. An effective camera–marker detection method was developed to follow the leader, which was controlled by an operator. A solution for enhancing image contrast that involved using the histogram method, offsetting vehicle roll angle, and estimating and smoothing the camera observation using the least-squares method ensured a stable and accurate monocular vision system that was able to estimate the relative position between the leader and the follower vehicles with high accuracy. A feedback control rule and a PID controller were also developed and exhibited good performance for linear and parallel leader trajectory tracking. The estimation and smoothing of the camera observation data reduced camera noise and yielded relative positional information between the leader and the follower vehicle with high accuracy. As a result, a stable velocity and steering angle of the follower vehicle and high accuracy of the trajectory tracking was established. Thus, a low-cost, reliable navigation system for a leader and follower vehicle tracking system was demonstrated. In further research, the leader should be converted to a remote control unit to make it unnecessary for the operator to be on-board the leader vehicle. Additionally, to overcome the limitations of the prototype leader-follower system, such as guidance in the adverse climatic conditions are required to consider for agricultural operations.

## Nomenclatures

The following nomenclature was used in this manuscript:

D: Relative distance between the leader and the follower, m	$q_{Fit}$: Vector of fitted current relative distance and relative angle using the stored *n* points of observation data
β: Relative heading angle between the leader and the follower	$q_{Est}$: Vector of the current relative distance and relative angle
α: Orientation angle of the leader relative to the follower	q(i): Vector of stored $i_{th}$ observation
H: Side length of squares on marker, m	$q_{E\_1}$: Vector of distance between current observation and last observation
L: Interval between square centers, m	$q_{E\_2}$: Vector of distance between current observation and fitted observation
$\alpha_{s}$: Angle between square center and camera optical axis	$q_{Th}$: Vector of threshold values
$\alpha_{En}$: Angle between optical axis and the follower centerline	$x_{req\_L},y_{req\_L}$: Required position of the follower in the leader-based local coordinates, m
h: Height of squares in the image plane, m	$\theta_{req\_L}$: Required heading angle of the follower in the leader-based local coordinates
$f,\;f_{x}$: Camera focal length	*l*: Length of vehicle wheelbase, m
$c_{x}$: Shift of camera optical axis	$l_{c}$: Length from the follower rear wheel axial center to the control point C, m
γ: Roll angle of camera around its optical axis	$d_{01}$: Required relative distance between the leader and the follower, m
$x_{cn},y_{cn}$: Coordinate of square centers under image coordinate system, pixel	$\Phi_{01}$: Required relative heading angle between the leader and follower
$X_{CN},Y_{CN},Z_{CN}$: Coordinate of square centers under camera coordinate system, m	$x_{c\_L},\;y_{c\_L}$: Local position of the control point *C* in the leader-based local coordinates, m
$X_{HN},Y_{HN},Z_{HN}$: Coordinates of square centers with respect to the horizontal surface, m	$\theta_{c\_L}$: Local heading of the control point *C* in the leader-based local coordinates
$X_{VN},Y_{VN}$: Coordinates of the square centers in the follower-based local coordinates, m	$x_{e\_c}$: Control point C-based lateral tracking error, m
$x_{l\_F}$, $y_{l\_F}$: Local position of the leader based on the follower, m	$y_{e\_c}$: Control point C-based longitudinal tracking error, m
$\theta_{l\_F}$. : Local heading angle of the leader based on the follower	$\theta_{e\_c}$: Control point C-based heading tracking error
q(n): Sequence of stored observation data	δ: Steering angle of the follower vehicle
$q_{C\_obs}$: Vector of current camera observed data	v: Velocity of the follower vehicle, m s^−1^
